# Is there any role for community involvement in the community-based health planning and services skilled delivery program in rural Ghana?

**DOI:** 10.1186/1472-6963-14-340

**Published:** 2014-08-11

**Authors:** Evelyn Sakeah, Lois McCloskey, Judith Bernstein, Kojo Yeboah-Antwi, Samuel Mills, Henry V Doctor

**Affiliations:** Social Science Unit, Navrongo Health Research Centre, Navrongo, Upper East Region, Ghana; Department of Community Sciences, Boston University School of Public Health, Boston, MA USA; Department of Global Health, Boston University School of Public Health, Boston, MA USA; Health, Nutrition, and Population, Human Development Network, The World Bank, Washington, DC USA; Integrated Programme and Oversight Branch, Division for Operations, United Nations Office on Drugs and Crime, Abuja, Nigeria

**Keywords:** Community-based service delivery, Ghana, Skilled birth attendance, Community participation, Community health workers

## Abstract

**Background:**

In Ghana, between 1,400 and 3,900 women and girls die annually due to pregnancy related complications and an estimated two-thirds of these deaths occur in late pregnancy through to 48 hours after delivery. The Ghana Health Service piloted a strategy that involved training Community Health Officers (CHOs) as midwives to address the gap in skilled attendance in rural Upper East Region (UER). CHO-midwives collaborated with community members to provide skilled delivery services in rural areas. This paper presents findings from a study designed to assess the extent to which community residents and leaders participated in the skilled delivery program and the specific roles they played in its implementation and effectiveness.

**Methods:**

We employed an intrinsic case study design with a qualitative methodology. We conducted 29 in-depth interviews with health professionals and community stakeholders. We used a random sampling technique to select the CHO-midwives in three Community-based Health Planning and Services (CHPS) zones for the interviews and a purposive sampling technique to identify and interview District Directors of Health Services from the three districts, the Regional Coordinator of the CHPS program and community stakeholders.

**Results:**

Community members play a significant role in promoting skilled delivery care in CHPS zones in Ghana. We found that community health volunteers and traditional birth attendants (TBAs) helped to provide health education on skilled delivery care, and they also referred or accompanied their clients for skilled attendants at birth. The political authorities, traditional leaders, and community members provide resources to promote the skilled delivery program. Both volunteers and TBAs are given financial and non-financial incentives for referring their clients for skilled delivery. However, inadequate transportation, infrequent supply of drugs, attitude of nurses remains as challenges, hindering women accessing maternity services in rural areas.

**Conclusions:**

Mutual collaboration and engagement is possible between health professionals and community members for the skilled delivery program. Community leaders, traditional and political leaders, volunteers, and TBAs have all been instrumental to the success of the CHPS program in the UER, each in their unique way. However, there are problems confronting the program and we have provided recommendations to address these challenges.

## Background

In Ghana, between 1,400 and 3,900 women and girls die each year due to pregnancy related complications [1, 2]. In 2013, approximately 3,100 women in Ghana died from pregnancy-related complications [3]. An estimated two-thirds of these deaths occur in late pregnancy through to 48 hours after delivery [2]. Recent statistics point to a maternal mortality ratio (MMR) in Ghana of 380 deaths per 100,000 live births. This MMR is high when compared with that of other sub-Saharan African countries such as Namibia, which has a MMR of 130 deaths per 100,000 live births but is lower than the sub-Saharan African regional estimated average of 510 maternal deaths per 100,000 live births [3].

In sub-Saharan Africa, only half of women delivered with the assistance of skilled attendants [4]. In Ghana, skilled delivery rate is slightly higher (68 percent); but there are rural–urban disparities. In 2011, only 54 percent of rural women were delivered by skilled attendants at their birth compared to 88 percent of urban women [5]. In rural areas of the Upper East Region (UER) which is the focus of this paper, the level is higher (67 percent) than in other rural areas in Ghana [5].

Experts agree that access to skilled attendants at birth (doctors, nurses, midwives) is one way to decrease maternal deaths, and such access should be available to women in rural areas as well as urban areas [6]. Since 2000, the government of Ghana has had an innovative program to promote primary health care in rural communities—the Community-based Health Services and Planning (CHPS) Program. In 2005, the Ghana Health Service piloted a program that involved training Community Health officers (CHOs) as midwives to address the gap in skilled attendance in rural UER. Community participation was an integral part of CHPS and the CHO-midwifery pilot project from its inception, and results are promising [7].

Community participation is a significant part of health service delivery in developing countries. Community health volunteers have been used elsewhere to encourage community involvement and to compensate for severe shortages of health professionals [7–9]. The 1978 Alma-Ata Conference reiterated the goal of “Health for All by the Year 2000” and approved primary health care (PHC) as key to achieving this goal [10]. Social intervention have demonstrated the active engagement of communities in primary health care that resulted in improved health [11–15].

Evidenced-based interventions have demonstrated the significant role communities played in PHC that resulted in improved health [11–16]. For instance, the Danfa comprehensive rural health and family planning project demonstrated how village health workers improved health services in rural Ghana [16]. The Bamako Initiative which was implemented to increase access to PHC by engaging village committees in health delivery management resulted in improved child health [11]. Haines et al. cited how field interventions have established community health workers contributions to high child survival coverage and other health programs and suggested evaluating the role community participation played in increasing coverage of essential interventions [14]. These programs have demonstrated the effective role of communities in improving health service delivery and pointed out the need for rigorous evaluation to determine the level of community participation and appreciate the contributions of communities to health intervention programs.

A study on the use of safe motherhood promoters to improve patronage of health facilities for skilled delivery and antenatal care in Tanzania showed an increase in skilled deliveries and antenatal attendance after a successful program implementation [12]. In rural Burkina Faso, the use of health professionals and community members in providing maternal and child care significantly increased institutional births and reduced maternal and perinatal deaths [17]. Other studies have demonstrated that the combined efforts of communities and CHOs in the Community Health and Family Planning (CHFP) project in northern Ghana revealed a 15 percent reduction in fertility, equivalent to one birth per woman in the general population [9]. Doctors established that the shared contribution of community health volunteers and CHOs to health delivery care in rural northern Ghana motivated communities’ preference for smaller families through changes in reproductive behavior [15]. The success of the CHFP project led to the implementation of the CHPS Program [7] and later the introduction of the midwifery program to bring maternal health services to the doorsteps of women and their families.

The CHPS program, an initiative of the Ghana Health Services launched in 2000, was designed to increase access to health care and family planning services in rural areas of Ghana [7]. A CHPS zone has a population of 3,000 to 4,500 people–covering two to three unit committees of the District Assembly [18]. All sub-districts are divided into zones and these zones are based on the local government of decentralization, where the District Assemblies and unit committees cover a population of approximately 1,500 [18]. From its inception, community participation was a significant component of the program. Local communities provided land and labor for building CHPS compounds and community health volunteers undertook health education and management of minor ailments. CHO-midwives partnered with traditional birth attendants (TBAs), community health volunteers and other community members to provide skilled delivery in rural areas. As the program seeks to ensure that all women in remote villages have access to skilled attendants during and after delivery, strong partnership between TBAs and health professionals and between all community members and the CHO-midwives are essential [7].

A CHO-midwife is an auxiliary nurse with a two-year training in basic nursing and another two-year training in midwifery by WHO standards to provide basic health services including skilled delivery care in rural areas [7]. Results of the CHPS program showed that nearly 80 percent of women were supervised by skilled attendants at birth in CHPS zones from 2009 to 2012 in three districts of the UER [19]. Other studies have also shown the positive impact of CHPS on maternal and child health in Ghana [20–22].

This study examined the extent to which community residents and leaders participated in the skilled delivery program as part of the CHPS program, and the specific roles they played in its implementation and effectiveness.

## Methods

### Study setting

The study was conducted in the Kassena-Nankana East (KNE), Kassena-Nankana West (KNW), and Bongo districts of UER of Ghana. The UER is situated in the north-eastern corridor of the country and share borders with Burkina Faso to the north, and Upper West Region to the west, Togo to the east and Northern region to the south. The estimated population from the 2010 census was 1,046,545. The settlement pattern is highly dispersed in 911 communities. The KNE district had an estimated population of 109,944 [23] whereas the KNW district, newly carved out of the Kassena-Nankana district in UER, had an estimated population of 70,667 in 2010. Bongo district’s 2010 estimated census population was 84,545 [23].

In rural UER, households are grouped into extended family units or compounds, each headed by a male patriarch. Compounds are located far from each other, but the people have a communal life style, where support systems exist for community development activities [24]. Lineage customs, religious practices, marriage patterns, and other social characteristics of the population are traditional, but changes such as construction of roads, schools, hospitals/clinics, modern houses are gradually taking place. The three districts share a homogeneous social and cultural system [24]. The region has one regional hospital located in Bolgatanga, the regional capital; 5 district hospitals; 26 health centers and 35 clinics run by the government health service, mission institutions and the private sector and 200 functional CHPS compounds [23].

We obtained ethical approval from the Navrongo Health Research Centre and the Boston University (BU) Institutional Review Boards (BU IRB reference number H-31245). We also obtained written informed consent from participants before they took part in the study.

### Study design and methods

We employed an intrinsic case study design with qualitative methodology. We conducted in-depth interviews (IDIs) with key informants such as chiefs, TBAs, community health volunteers, women leaders and elders: Both trained and untrained community health volunteers and TBAs were included in the interviews. We also interviewed health professionals (CHO-midwives, District Directors of Health Services and the CHPS Coordinator) to (1) assess the extent to which communities know and use the skilled delivery services, and (2) identify contributions community residents made to the program and to explore successes and challenges of implementing the program. We also looked at the Navrongo Health and Demographic Surveillance System (NHDSS) data to determine trends in unskilled deliveries in the KNE and KNW districts.

### The Navrongo health and demographic surveillance system

The NHDSS is located in the Kassena-Nankana districts of northern Ghana; the NHDSS was established in 1992 by the Navrongo Health Research Centre (NHRC). The main objective of the Navrongo Demographic Surveillance System (NHDSS) is to maintain a longitudinal follow up of the population dynamics of the Kassena-Nankana district (152,000 people in 32,000 households.) Every 4 months the NDSS collects and computer processes routine information on pregnancies, births, morbidity, deaths, migration, marriages and vaccination coverage. NHRC also conduct biomedical and socio-economic studies and trained volunteers to routinely report key events, such as births and deaths as they occur in their locality and the verbal autopsy system for determining the probable causes of deaths that occur in the community [25].

### Sampling and sample size

A total of 79 CHPS compounds were located in the three districts and of that a total of 25 CHPS compounds had CHO-midwives. Our target population has 25 CHO-midwives working in CHPS zones in the three districts: Ten CHO-midwives in the KNW district, eight in the KNE district and seven CHO-midwives in the Bongo district of the CHPS zones. We randomly selected 12 from the list of 25 CHO-midwives to participate in the IDIs: 4 CHO-midwives from each of the three districts. And out of the 12 CHO-midwives selected for the interview, 10 CHO-midwives participated. The other two participants were not available for interview after several attempts to meet and interview them. Three District Directors of Health Services and the Regional Coordinator of the CHPS program were purposively selected because of their role in the CHPS program in the study areas. Fifteen community stakeholders were recruited for the in-depth discussions in the three districts. We recruited the community stakeholders through speaking with key community members such as opinion leaders, chiefs, and elders. We gathered names of those in each group who were most knowledgeable about the program. Among those identified as potential respondents (7 Chiefs, 10 Elders, 8 TBAs, 10 Community health volunteers and 8 Women Group Leaders), we randomly selected one in each group within each district to approach for an interview. In each district, we included five community stakeholders and a total of 15 stakeholders were interviewed. In all, 29 key informants (10 CHO-midwives, 3 District Directors of Health Services, 1 Regional Coordinator of the CHPS program and 15 community stakeholders) were interviewed.

### Training of data collectors

We recruited two university graduates who are also experienced research assistants and trained them to conduct the IDIs. The training included lectures, demonstration and practice. They were trained to moderate the interviews and use the interview guide in local languages of the three districts. The research assistants were coached to ask questions, probe for more answers and prompt respondents for clarifications on issues related to the interview. The training lasted for a week after which the interviewers were sent to the field for the data collection. The interviews were recorded on an audiotape. An experienced transcriber, who was not part of the interviewing team, translated and transcribed the data from the audiotapes before analysis of the transcripts.

The interview formats focused on the extent and type of participation by community stakeholders in the skilled delivery program, successes and challenges of the program from their perspectives.

We pre-tested the interview guides in communities of the three districts excluded from the study, but have similar characteristics, in order to improve the relevance and appropriateness of the questions. The pre-testing was a learning session for the research assistants to improve their interviewing skills, and we revised the guides appropriately after the pre-test. Data were collected from January 13, 2012 to March 31, 2012.

### Data analysis

The analysis of narrative data on similar topics from multiple sources allows for comparison of perspectives and triangulation of our findings across sources. Members of the team (the Principal Investigator and the two research assistants) began the analysis by reading all transcripts multiple times and discussing broad themes that emerged across respondents and areas of inquiry (community participation and benefits/challenges). The team developed a coding scheme that reflected these areas and the sub-themes within each, and proceeded to code each transcript using the qualitative data software (QSR NVIVO software version 8). We produced reports on each of the broad and specific themes, which allowed us to synthesize key findings and compare responses within and between groups (e.g., community stakeholders and health professionals). The broad and specific themes included knowledge and contribution by community leaders: contributions of TBAs, community health volunteers, traditional and political leaders; each stakeholder groups’ perspectives on the successes and challenges of the program in key areas, such as promoting the use of skilled attendants at birth, the perceived contribution of program in preventing complications and deaths, and challenges such as long distance, lack of transportation, insufficient infrastructure, attitudes of health professionals and inadequate medicines.

## Results

### Knowledge and contribution by community leaders

Community leaders were generally informed about any new program before they became involved in implementing the activities. Likewise, community leaders with whom we spoke all knew about the CHPS program and were in close consultation with health professionals in the planning and construction of CHPS compounds. They also helped to implement the program and often sought skilled delivery care and treatment for minor ailments from the health professionals. The community leaders described their involvement in the CHPS program:

*…*“*Yes, I know about the existence of the small hospital [CHPS compounds]. When the health professionals first came to Kandiga chief’s house they called all the elders; all sectional elders from Nindagsi, Kruba, Bembisi---in fact elders from all the sections to meet at the chief’s house and they told us how they wanted to establish the small hospitals [CHPS compounds] to help us. They did not just come to start it all alone; they did it in consultation with the community members, the chief, his elders, and opinion leaders before they started”***(Community health Volunteer, KNE District)**

### Contributions of women, family and other community members towards the skilled delivery services

CHO-midwives and community health volunteers confirmed how community members participated to ensure that pregnant women received skilled care: For instance, community members referred or accompanied pregnant women to the CHPS compounds for skilled delivery services. Respondents gave examples of how they work for the program.

*……*“*You know everything is about money so when a pregnant woman has a problem and may not have money, we have a brother here in the community when such situations arise; we contribute money, go to the man and ask for help. We buy petrol and put it in his vehicle to take the woman to the big hospital. The man is called [name withheld]. Anytime there is a problem in the night, wherever a motorbike or a bicycle cannot go to and we cannot also walk there, we run to him. The community members also contribute some money for him to take the woman to the hospital.”***(Community health Volunteer, KNW District)**

*……*“*We asked the young men in this community to mold bricks because it is their wives who go there to deliver. They molded the bricks to build rooms for the nurses. When they were going to start with the building itself, the community members met and contributed money little by little towards putting up the building”***(TBA, KNE District)**

*…..*“*Yes, the community contribution is there because if the midwife is there and they are not patronizing the services then there is no need putting a midwife there. Even getting a place to put up the facility is from the community. The communities are also actively involved in the skilled delivery program.”***(District Director of Health Services, Bongo District)**

The stakeholders revealed that some women contributed to the skilled delivery program by seeking skilled care. The women’s groups also assisted the midwives in their household chores.

*…..*“*I don’t even wait for them to tell me what to do because I’m carrying the pregnancy and I know the consequences of delivering with someone who is not a health professional. Therefore, if there is any problem I go to the hospital.”***(Women’s Group Leader, KNW District)**

*….*“*When the nurse first came, the women were divided into groups, one group would fetch water for her and another would sweep round the compound so it shows that the nurse does not work alone, she works with the community members.”***(Women Group Leader, KNE District)**

However, a traditional leader reported that some rural communities in the KNE district do not have women’s groups that are actively involved in the skilled delivery program. The leader, therefore, suggested that communities should volunteer to assist the midwives and existing men and women’s groups should be encouraged to get involved in the program. A key informant stated:

*…*“*We do not have women’s group helping the midwife and other nurses in this community. We have to choose women who should be trained to help the midwife and the other nurses in the CHPS compound. We also have to talk to the women’s groups’ leaders to urge their members to help the health staff because it is important for them to be involved to get the work going.*” **(Chief, KNE District)**

### Donation of land and other logistics and provide communal labor towards the building of the CHPS compound

Rural communities in UER are no longer mere recipients of health services, but they also donate to the CHPS program. Key informants reported that communities provided land, labor, funds, and other logistics for the building of CHPS compounds. The following quote from a community stakeholder illustrate how they described community contributions to the program:

*….*“*When they were going to build the CHPS compound the district assembly only brought a tipper truck and the community members came out and collected sand for the building of the project, the community members donated the land free of charge and they picked stones for the project. During the construction, digging of the foundation, fetching water; they call it communal labor, whenever they announced all members of the community came out to work at the small hospital [CHPS compound].”***(Community health Volunteer, KNW District)**

### Contributions of community health volunteers to the skilled delivery program

Respondents stated that communities participated in weighing children, providing health education, and treating minor ailments. They also referred or accompanied pregnant women to the CHPS compounds for skilled delivery care. In a stakeholder’s own words:

*..…*“*I am involved in weighing in the CHPS compounds or during outreach programs. We hold talks with the women about the importance of skilled delivery care and we often refer or accompany pregnant women to the small hospital [CHPS compound] for the skilled delivery services.”***(Community health Volunteer, KNW District)**

### Contributions of TBAs to the skilled delivery services

Health professionals and community stakeholders affirmed that TBAs provided health education on the importance of skilled attendants at birth to women. They also referred or accompanied their clients to the CHPS compounds for skilled delivery services. The statement of a TBA captures this transition in the role of TBA’s in birthing:

*…..“I help the nurses by saving women in labor in the night by accompanying them to the CHPS compound for delivery services. During the day, I also take any woman in labor to the small hospital [CHPS compounds] to deliver. If a woman is not able to deliver they take the woman and I on a motorbike to “Fari yeri” [Catholic Mission Health Centre] to deliver her. “***(TBA, KNE District)**

Although some TBAs referred their clients to the CHO-midwives for skilled delivery services, community residents reported that others still provided delivery services, but when some of them detected a complication, they quickly referred or accompanied the women to the CHPS compounds for professional care. Community stakeholders reported that some TBAs provide delivery services for traditional, cultural and financial reasons. Most respondents described the work of the TBAs, which included delivery and referral, as illustrated in this quote:

*....*“*I have delivered so many women and I have referred pregnant women with complications to the small hospital [CHPS compound] for care. I have also talked to pregnant women to go to the small hospital for weighing and also for delivery. Many women now go there for weighing and delivery services.”***(TBA, KNW District)**

Some stakeholders reported that a few TBAs have supernatural powers, which could help women deliver easily. Thus, women sometimes prefer to be supervised by these TBAs during birth. In addition, cultural or distance factors are reasons for husbands preventing their wives from delivering in a health facility.

*…..*“*There is a man, who is a TBA; it is a tradition in their family since time immemorial. There is juju’ [a form of magic] in their family and if a woman cannot get pregnant goes to that house, they can treat her to get pregnant and deliver a child. Any woman who is in labor and cannot deliver, if they call that man he will come and deliver her.”***(Elder, Bongo District)**

*…..*“*Some husbands do not allow their wives to deliver in the hospital. They prefer that the women deliver at home because of lack of means of transportation or the tradition does not permit them to deliver in the hospital.”***(Women’s Group Leader, Bongo District)**

### Incentives for trained TBAs and volunteers

Respondents reported that CHO-midwives gave soap or money to trained TBAs and volunteers who referred or accompanied pregnant women to the CHPS compounds for supervised delivery. Communities also accorded TBAs and volunteers “respect” and “recognition”, which is an incentive to them. These issues are highlighted by a key stakeholder in the following excerpts:

*…*“*I have used a percentage of the delivery fee to purchase soap and the soap is here; when a TBA or a volunteer accompanies a woman to this place to deliver we give them the soap, they are happy and so, they continue to refer the women.”***(CHO-Midwife, KNE District)**

However, a few volunteers complained that they were not compensated for their services. The Ghana Health Service only compensates trained community health volunteers, so those who do not fall within this category are not rewarded.

*…..*“*In fact, on many occasions, no one thinks about we the volunteers, and that makes us drag our feet when we are working. I have done this voluntary work for the past ten years. I am only doing it for my community. No one comes to see us [nobody motivates them] and the women do not have the money to assist us the volunteers. This does not help the work in anyway. We help to send pregnant women to the CHPS compound to deliver and at times we call the nurse to a compound to deliver a woman in labor and so they should motivate us.”***(Community health Volunteer/Women’s Group Leader, Bongo District)**

### Contributions of traditional leaders: paramount and sub-chiefs

The discussants stated that traditional leaders were involved in the program activities by providing health education in the communities to ensure that every woman received skilled care at birth. The chiefs donated land and provided labour for the building of the CHPS compounds. They also put bylaws to punish women and their families who refused to deliver in health facilities and the penalty includes payment of a sheep:

*…*“*If they have any problem that concerns the community and there is need for me to help them get the discussions going, I do it. Especially meetings if they inform me, I also ask the smaller chiefs to announce to their people to come out on the stipulated day to hear what the nurses have for us. Yes, I do the announcement and then ask the smaller chiefs to also announce to get the information to every section.*” **(Chief, KNW District)**

*…*“*We held a durbar here and the chief [Name withheld] said any woman who delivered at home will be sanctioned. So the women are very careful not to deliver without the supervision of the trained midwife.”***(CHO-Midwife, KNW District)**

### Contribution of the district assemblies to the skilled delivery program

The District Health Administration had collaborated with the District Assembly for electrification/water source for CHPS compounds without lights and water. Community leaders and health staff stated that the District Assemblies built some of the CHPS compounds and assisted in acquiring land and other logistics such as tipper trucks to carry sand, stones, equipment and logistics for the building. Occasionally, the assembly members assisted in organizing the communities for health programs. This quote illustrates what we learned:

*…*“*Let me say that I have been able to convince the District Assembly and they have also seen the need to improve on the structure. They have constructed a CHPS compound at Nyangua; there is antenatal care, delivery and rest rooms separate from the Outpatient Department. They have also provided staff accommodation; so it is a big structure. Now they have finished and handed over to us. They have put the same structure at Korania and they are in the process of handing over to us.”***(Health Official, District Health Management Team, KNE District)**

### Success in promoting skilled attendants at birth

Community stakeholders indicated that most pregnant women no longer deliver at home through the collaborative efforts of community members and health professionals. A significant impact of the program is that community members do accompany or refer pregnant women to CHO-midwives for skilled delivery services. Both the health authorities and the community members attested that they collaborated to ensure that every woman receives skilled care at birth in rural areas.

*…*“*In fact for the past four years, any pregnant woman who is in labor does not go far to deliver. Women now go just nearby to deliver; but before now any woman who was in labor had to get a vehicle to Bolga. Some women even delivered on the way because of lack of the means of transportation to get to Vea quickly. Throughout rainy season pregnant women could not go to Vea so they had to go to either Bolga or Bongo to deliver. But now women do not go far to deliver, they give birth in the quarters [CHPS compound] here.*” **(Women leader-Bongo District)**

*…“At first when a woman was in labor, we got men or women here who would try by all means to bring the baby out, but after the baby came they would not know how to deal with problems associated with the delivery. With the nurses here after they have delivered a woman, they allow her to rest in the facility till the following day to see if she has any problem. It is now better than when our own people were assisting the women to deliver. TBAs and volunteers help the nurse to get the pregnant women to the health facility for skilled delivery. We no longer have so many problems; it is so helpful since they go to the nurses to deliver.”***(Community health Volunteer, KNE)**

The Kassena-Nankana Health and Demographic Surveillance System data confirmed the decline in home deliveries in the KNE and KNW districts from nearly 70 percent in 2003 to less than 40 percent in 2009.

### Success in preventing complications or deaths

One important reason for implementing the skilled delivery program is to ensure that every woman delivers safely. In the three districts, community stakeholders emphasized that a huge benefit from the skilled delivery program was that women no longer die from pregnancy related causes. As community health volunteers expressed it:

*…..*“*E!! The biggest benefit is, women used to die during labor, but now because of the help they get from this CHPS program, women no more die during delivery because once the nurses get there, by God’s grace the baby comes out without any problem. It is a benefit, they save lives; they also hold discussions with the women on how to keep themselves until they deliver. It is very beneficial.”***(Community health Volunteer, KNW district)**

“*Since the trained midwife delivers women in this community, the women are healthy; the nurse gives pregnant women medicine so they deliver safely without any problem. At first it was not so, pregnant women used to suffer during delivery, but now if a woman delivers, they give her and her baby medicine so they are healthy.”***(TBA, KNE District)**

### Challenges facing the skilled delivery program: long distance, transportation and medicines

Notwithstanding the successes, the skilled delivery program is still confronted with many challenges. Respondents cited lack of transportation as a key barrier to referring clients to the CHPS compound, health centre or the district hospital for care. Some community stakeholders mentioned long distances to CHPS compounds as a reason for their non-involvement in the skilled delivery program. A key informant stated:

*…* “*It is a problem here during rainy season, how to cross the river to this place is a big problem; even if you are going to Bongo, there is another river at Balungo. When the river gets full we are cut off. It is only during the dry season we are free. That is why the facility has been sent there. By God’s grace we will construct one here. If you want to travel from here; you will sweat [struggle] before you go. There are no vehicles; Bongo which is even bigger has only one vehicle which sometimes breaks down. Our road is also not good. That is the problem so if every community has a small hospital [CHPS compound] it will be helpful to people. Then if it is beyond the nurses they can refer to the big town [District hospital].”***(Chief, Bongo District)**

We found mixed reports about the availability of needed medicine in the CHPS compounds. While some community stakeholders explained that there were no medicines in the CHPS compounds, others said that some CHPS compounds have no problem with medicines. A health official confirmed that the CHPS compounds had regular supply of medicines.

*….*“*In fact in this small hospital [CHPS compound], at times getting medicines is a problem; even if you have a health insurance identity card they will tell you there is no medicine. Even if you go to the big hospital [District hospital] in town they will tell you there is no medicine. They will tell you to go to a drug store and when you get there, the medicine is so expensive. But we thought having the insurance card would help us, it is not so. So I think if they have medicines all the time it will help us.”***(Chief, KNE District)**

*…..”We could not get medicines when we fell sick, but now we get medicine right here. Our pregnant women had problems, now any woman who is pregnant goes to the small hospital [CHPS compound] for weighing and takes her medicine until she delivers. No more problems.”***(Elder, Bongo District)**

*….*“*Now the CHOs have no problem with drugs because we have a scheduled delivery where the regional medical stores go round them with drugs. They (CHOs) provide the clients with the necessary drugs and then claim from the National health insurance authority.”***(CHPS Coordinator, Regional Health Directorate)**

Respondents also complained of poor infrastructure and inadequate logistics for the skilled delivery program in CHPS zones.

*….“They cannot work effectively in the night here because there is no electricity, we have no lorry here, when we have a problem, it is Bongo we have to walk to, to get transportation. With the hospital, I cannot say it is too small; but the fact is that, Adaboya [the community] is big. So we want a bigger one [CHPS compound] than this one; the delivery room can take only one woman at a time. If there are four women in labor they cannot all go in to deliver. Others will have to wait outside.”***(Elder, Bongo District)**

*….“You know as at now the structures put up are not service delivery friendly because they made it in way that they will only be doing homes visiting and just treatment of minor ailments. Now that we are adding this delivery, you know wherever there is a midwife you need a room for prevention from mother-to-child transmission* (*PMTCT*) *of HIV, counselling, and testing and everything, At least we need adequate structures to be put in place so that they can carry out the right services that are expected.”***(District Director, Bongo District)**

The CHO-midwives reported that in some traditional settings, women still do not access health care because of customs and taboos that forbid them from visiting health facilities. The midwives said in the process of convincing families to send pregnant women to health facility for care, for example, delays occur and that leads to death of the pregnant women or babies, as the following quote illustrates:

*…..“Yes, in fact there are some houses around here; they say they taboo hospital and so when their women are even pregnant, they do not attend clinic. Those who even attend; one ever came, we realized that the lying of the baby was not normal. We asked her to go and do scanning and she refused. When she came into labor too, she came to the CHPS compound alright but when she came, she was almost full, she was referred to the hospital but because she delayed the baby died. When she came home they said because they taboo going to hospital made the child to die. When the woman was pregnant again, I followed her to go to clinic, but she refused to go because she had to talk to members of the family for permission. However, people also came in to talk to the family before she was allowed to attend antenatal clinic.”***(CHO-midwife, Bongo District)**

Some community stakeholders complained of the poor attitudes of a few nurses, which can ruin the reputation of all if not addressed:

*……*“*Some women complain that some nurses shout at them and you know it is not everyone who feels comfortable with that. You may be saying something good to someone but if you say hey!! The person’s heart will jump. So whatever you say to the person, you will only be singing. So the nurses should exercise patience. You know, a pregnant woman is like a hungry person; a hungry man is an angry man. So when a woman is pregnant, it makes her angry without any reason. The nurses should exercise patience when they are explaining things to them.”***(Community health Volunteer, KNW District)**

### Lessons learned from community contributions to the skilled delivery program

We asked stakeholders about their views of the pilot program and lessons they had learned about community participation as implemented. First, they confirmed their strong belief that skilled attendants at birth would prevent maternal and infant mortality and morbidity. Second, community leaders stated that community members not be viewed as “empty vessels”, but rather partners in solving their health problems. They reiterated the critical ingredients for strong collaboration among stakeholders: respect, dialogue and cooperation. In their own words:

*….*“*I have learned a lot; when I was a child I saw how women suffered and died during pregnancy and now that the nurses are here, they save the lives of pregnant women.”***(Community health Volunteer, KNW District)**

*…..*“*I will say that the government has done well by sending trained midwives to the communities to provide skilled delivery care. I am no more worried when pregnant women are going to deliver because they will come back safely. We are thankful to the health authorities.”***(Elder, KNE District)**

*….*“*Through this program I have come to realize that communities could also contribute to their health needs. I think community involvement is a key strategy in engaging communities to solve their own problems. We are ever grateful to the Ghana Health Service for this idea of bringing trained midwives to assist our women during delivery”***(Elder, KNW District)**

*…..*“*Community members are not “empty vessels” so they should be encouraged at all times to contribute to their health or development needs. The key issues are respect, dialogue and cooperation on the part of communities and health authorities to guarantee community involvement in health programs.”***(TBA, KNE District)**

## Discussion

This study reinforced and elaborated on findings of other studies in sub-Saharan Africa (and elsewhere) showing the significant contribution community members make to community health programs [11, 12, 17, 26–28]. Several authors from South Africa, Burkina Faso, Tanzania, and Peru have written previously about the pivotal role communities play in increasing skilled attendants at birth and reducing maternal deaths in rural areas [11, 12, 17, 26–28]. Haines et al. demonstrated how community health workers contributed to high child survival coverage and other health programs that improved health outcomes [14].

### Contributions of TBAs

TBAs have been an integral part of the health system in Ghana. TBAs were initially trained to provide delivery services in rural communities to augment the work of the few skilled attendants in those settings [17]. The introduction of the CHPS program in rural communities strengthened the collaboration between TBAs and health professionals for the former to refer their clients for skilled attendance at birth. Our findings revealed that TBAs referred or accompanied their clients to CHPS compounds for skilled delivery services. Our results are consistent with previous studies that revealed that this kind of collaboration resulted in TBAs referring or accompanying many more pregnant women to health facilities for skilled delivery care [29]. This study also demonstrated that some TBAs would only refer their clients when there are complications. Yousuf et al. also reported that a trained TBA would refer a pregnant woman for skilled delivery care after an abnormal presentation, prolonged labor, obstructed labour, and excessive blood loss [30]. However; our study is in contrast with a study that found that training of TBAs was not associated with client referrals [30].

The Ghana Health Service is sending trained midwives to rural communities and the roles and responsibilities of TBAs are being redefined. In many instances, community members had to contribute to transport the TBAs and the pregnant women to the CHPS compounds for skilled care, and that probably motivated the TBAs to refer or accompany their clients for the skilled delivery services. Also, the “respect” and “recognition” community members accorded TBAs for their role in the skilled delivery program might have served as an incentive to them. Likewise the incentives the CHO-midwives gave to the TBAs could also be a motivational factor for referring their clients for skilled delivery. Communities’ accessibility to the CHPS compounds and availability of trained professionals undoubtedly serve as an incentive for the TBAs referring or accompanying their clients to the health facility for skilled delivery services. However, some TBAs continue to practice for a living or for traditional, cultural and financial reasons.

### Contributions of community health volunteers

Much previous research has underscored the contributions of community health volunteers to health programs [8, 9, 11–13]. In Ghana, too, community volunteerism has been an essential part of health systems over time [8, 9, 11]. Our findings indicate that volunteers took part in a range of health activities embedded in the CHPS-based CHO-midwifery program, including the weighing of children, drug administration for minor ailments, health education as well as referring or accompanying pregnant women to the CHPS compounds for skilled delivery services. The basic criteria for selecting volunteers were their good attributes that included good character, spirit of voluntarism, diligence, trustworthiness and honesty. Selecting the right people to occupy the volunteer positions likely contributed to the critical role the volunteers played in promoting skilled attendants at birth in rural areas. Incentives to volunteers offered by CHO-midwives most likely contributed to their active role in referring or accompanying their clients to health facilities for maternity services.

The supervision of volunteers is also crucial to ensure that they operate within the scope of their expertise. The Ghana Health Service only identifies and rewards trained TBAs and community health volunteers, who refer or accompany pregnant women for skilled delivery, but the question is what happens to the other key players such as the untrained volunteers, TBAs, and older women and mothers-in-law, who also provide delivery services in rural communities? It is necessary for health professionals to identify all stakeholders, who provide the services and involve them in educating pregnant women to seek skilled delivery care. These stakeholders, if identified and motivated, can be “agents of change” by actively participating in the skilled delivery program. Sustaining the interest of these key players is a key challenge if the program continues and is disseminated to other regions.

### Incentives for community health volunteers and TBAs

Attempts have been made to motivate trained TBAs and health volunteers for their services in rural communities. Our results show that health volunteers were delighted that community members recognized and respected them for their contribution to the skilled delivery program. In almost all the communities, the CHO-midwives used a percentage of funds generated from the deliveries to purchase soap to motivate women who sought skilled delivery and for trained TBAs and health volunteers, who accompanied pregnant women to the CHPS compounds for skilled delivery care. In some communities, volunteers were given money as incentives for referring pregnant women for skilled delivery services.

Incentive schemes have been documented as effective strategies to inspire motivation and performance of health workers in the health system in Ghana and elsewhere [31, 32]. The efficiency of public health services in Ghana has been linked to provision of incentives [33]. However, provision of incentives to community health workers is often challenging and unsustainable since the majority of volunteers often expect to be compensated even if they are located in poor and resource constrained communities [34]. Nevertheless, it is not all compensation that must be in cash or gifts. Communities can assist their volunteers in diverse ways, such as helping them on their farms or household chores, as long as the incentives are culturally appropriate.

### Contributions of traditional leaders

Chiefs and elders exercise considerable influence in their communities. They are heads of the traditional setup, hence arbitrate and supervise development programs in their areas of jurisdiction. The traditional leaders contributed significantly to execute the CHPS program by soliciting community support and cooperation for implementing the program. They also served as philanthropists by donating land and logistics for constructing the CHPS compounds, organizing community members for communal labor and contacting health authorities for assistance in building the CHPS compounds. Some of the traditional leaders also ensured that pregnant women delivered with skilled attendants; they did so by sanctioning women and their families who refused to deliver with the CHO-midwives: Families that violated the law were supposed to pay a sheep, but reports from such communities revealed that such sanctions had never been implemented because of the level of cooperation from community members. This indeed was not in compliance with community participation, but traditional leaders have power to institute bylaws to ensure the safety of their people. In the traditional settings, communities entrust their powers in the leadership to govern them. In most cases, the leaders informed and encouraged community members to attend meetings and that contributed to the active involvement of these traditional leaders and community members. In most instances, the traditional leaders initiated the activities of the CHPS program before other community members got involved. These findings corroborate earlier studies on antenatal care coverage and skilled attendance in rural Tanzania, which demonstrated the importance of traditional leaders’ approval for Maasai and the Watemi families to gladly seek services for pregnant women within the health system [35].

### Contributions of political leaders

The political leadership played a key role in implementing the maternal health program. The government introduced a policy of free medical care for pregnant women under the National Health Insurance Scheme, aimed at offering rural women the opportunity to seek skilled birth attendance. The majority of women in rural areas have already benefited from this initiative [36]. Also, the CHPS program relied heavily on the District Assemblies for support to construct the CHPS compounds and mobilize communities for health programs. The District Assemblies built some of the CHPS compounds for the CHPS program and provided tipper trucks to carry sand for constructing other CHPS compounds. They also constructed boreholes for clean and safe drinking water for the midwives and connected some of the CHPS compounds to the national electrification program. In many instances, the assembly members organized communities for health talks and also presided over the durbars. It is important that the government through the District Assemblies is investing in health care, which confirmed their commitment to the skilled delivery program. The study informed us about the importance of involving political leaders in the maternal health program and other health programs, and confirmed the need for the Ghana Health Service to continue to involve the District Assemblies in the design, implementation, evaluation and dissemination of health programs.

### Benefits

Health professionals in a collaborative effort with communities provided skilled delivery care to pregnant women to prevent injuries or death of women during delivery. Key stakeholders told us repeatedly that women no longer suffer complications or die during delivery in rural areas because of the presence of the skilled attendants coupled with community involvement. Findings from Burkina Faso also revealed that community mobilization could help reduce maternal and perinatal deaths [17].

Our findings also show that the training and deployment of CHO-midwives to rural areas together with community participation in the UER have contributed to improved skilled delivery access and utilization for rural women. Our findings are confirmed by a study of 407 mothers that revealed expanded skilled delivery care access and use since CHO-midwives were trained and deployed to work in CHPS zones [19].

We need further evaluation to understand the extent to which the CHO-midwifery program has led to recent improvements in maternal health in the UER in general, and more specifically, the particular contribution of community participation. However, our findings of this qualitative study allow us to argue that community mobilization is a significant strategy for improving maternal health in Ghana. The presence and services of the midwives in villages coupled with community active role in the program probably improved the use of skilled attendants at birth and averted many deaths that would have occurred in the hands of unskilled attendants.

### Challenges

The main barrier to skilled attendance at birth was accessibility. Although CHPS brings health services to the doorsteps of the people, some communities are very remote and far from the CHPS compounds, hence those affected recommended that CHPS compounds be built in their areas to help them access health care. Although the ideal is to establish a CHPS compound in every village, the cost involved in bringing that about makes the idea impracticable in the short run for the government. It is not practical to put a CHPS compound in every village, but it is possible to make health services available and accessible to most rural communities.

Skilled delivery care is free, but community members who reside far from the CHPS compounds cited transportation as a major reason for not accessing maternity services. The long distance to the health facilities and the absence of public transport in remote communities is a major obstacle to the use of professional delivery services. In rural northern Ghana, the common means of transportation is a bicycle, which is inappropriate for conveying pregnant women to health facilities for delivery care. In most rural communities both public and private vehicles are rarely available for those routes because they are not motorable. The absence of viable transport for pregnant women contributed significantly to the challenges communities faced in assuring universal access to health facilities for maternity services. Community members, who have motorbikes, sometimes manage to carry pregnant women to health facilities, but at a risk because those motorbike riders usually do not have safety measures to protect themselves and their passengers. Mills and colleagues (2007) also confirmed that lack of transportation is an obstacle to accessing health care in Ghana [37].

Some communities mentioned inadequate medicine, logistics and poor infrastructure in health facilities as further obstacles for the provision of efficient and effective services. It is crucial for the Ghana Health Service to guarantee a regular supply of medicines and logistics and adequate infrastructure if the CHPS skilled delivery program is to succeed. After all, rural communities can only build their trust in the system, if they may gain access to its services.

In addition to medicine, logistics, infrastructure, transportation challenges, community-sanctioned customs and taboos that prohibit visiting health facilities for care, stand in the way of skilled birth attendance for some pregnant women and their unborn babies. However, the key informants explained that such taboos are becoming outmoded, with more and more families seeking health care from CHO-midwives. A survey conducted in rural communities of UER confirmed our results that nearly all of them (99 percent) said there were no taboos that prevent women from giving birth with the assistance of a doctor, a midwife or a nurse (Evelyn S: *Utilizing the Community-Based Health Planning and Services Program to Promote Skilled Attendants at Delivery in Rural Ghana, unpublished PhD thesis,* Boston University School of Public Health).

Our findings also indicate that the attitude of some nurses towards their clients/patients is abysmal, which prevents some pregnant women from seeking skilled delivery services. Mills also reported the attitude of nurses as a major barrier for women accessing skilled delivery services (Mills S: *Utilization of Obstetric Services in northern Ghana: A Quantitative and Qualitative Assessment of Skilled Health Professionals at Delivery, unpublished PhD thesis* Johns Hopkins University School of Public Health).

### Study limitations

The research included a limited number of respondents, some selected based on the virtue of their position or role in the community. The small numbers and the uniqueness of the setting might not make the findings generalizable to other settings. On the other hand, the open-ended interview techniques allowed us to capture the views of the respondents in their own words. This study is focused on community participation in skilled delivery within the context of the CHPS program and might not be generalizable to other contexts because of the uniqueness of the design and implementation of the CHPS program in the UER. That said, our findings have salience to other similar programs in developing countries, geared towards reducing maternal morbidity and mortality in rural areas through the training of locally-placed community health practitioners as midwives. Social desirability and politeness biases may have been a possibility in this work, but study procedures and training protocols were designed to reduce these kinds of biases, and interviewers were university graduates who had no links to the delivery of health services. Respondent bias may have occurred since respondents were direct implementers of the skilled delivery program, and it may have been unlikely to identify stakeholders who were critical of the program. Despite these challenges, it is important to note that the people we interviewed were forthcoming with challenges and shortcomings of the program. Overall, the intervention in a remote and under resourced setting is perceived as a big leap toward improving people's health and access to health services.

## Conclusions

The CHPS program is one of many public health interventions of the Ghana Health Service that, by design and in practice, relies on community engagement for its implementation, widespread use, and ultimate success. Our study has shown that such mutual collaboration and engagement is possible. Community leaders, trained volunteers, TBAs, and other female and male members were all instrumental in unique ways to the success of the skilled delivery program in UER. Traditional leaders provided land and labor for the building of the CHPS compounds and collaborated with CHO-midwives to provide health education and information to rural communities. Political leaders also constructed some of the CHPS compounds and provided building materials for the building of others. The assembly members often presided on health programs at durbars. TBAs and community health volunteers collaborated with the CHO-midwives by referring or accompanying their clients to the CHPS compounds for skilled care at birth, and they provided health education to promote skilled attendance at birth. These volunteers were motivated by financial and non-financial incentives for their active engagement in the program. Overall, community members and health professionals appeared to demonstrate the key lessons of collaborative work: respect, dialogue and mutual cooperation. Nevertheless, the primary challenges that must be addressed include insufficient transportation; infrastructure weaknesses; poor attitudes among some health professionals toward their clients; inadequate drugs and other logistics; and to some extent, customs and taboos that prevent women from accessing maternal health services.

If the Ghana Health Services moves to scale up the CHO-midwifery program in CHPS zones beyond the UER, our study yields key recommendations to further enhance community participation in the program’s effectiveness:

- Health professionals should continue to intensify health education on maternal health in rural areas to enable all community members to embrace and participate in the program.
- Community health volunteers and TBAs should be engaged as “agents of change” to promote skilled attendants at birth, although their activities must be circumscribed and supervised to ensure that they do not act beyond their scope of services.
- The CHO-midwives should identify all TBAs, older women, mothers-in-law who still supervise deliveries and encourage them to refer or accompany their clients to the health centers or CHPS compounds for skilled delivery care.
- Volunteerism is free, but both the Ghana Health Service and community members could provide both financial and non-financial incentives to motivate community health volunteers and TBAs for their services. However, these incentives should be culturally appropriate without compromising the program. For instance, community members could assist the volunteers and TBAs on their farms and household chores to motivate them to continue to stay and work in rural areas. The communities could also give them certificates of recognition for the role they play in health service delivery.
- Community support systems should be instituted to assist pregnant women to seek maternity care on time; communities should collaborate with non-governmental organizations (NGOs), transport unions and individuals to institute transport systems to help convey pregnant women to health facilities for maternity services. NGOs should organize capacity building training for community members before these systems are set up.

In addition, our findings among community participants lead to several broader recommendations:

- The regional medical stores should ensure regular supply of drugs and other logistics to the CHPS compounds to guarantee the continuation of the program and build community confidence in the operations of the health system.
- The health professionals should be given regular in-service training to equip them to handle their clients and patients with respect and dignity.
- The health professionals should continue to embark on health promotion and education targeting families that taboo accessing modern health services to change their attitudes and practices.
